# MobiPhysio: A 2D video dataset of physiotherapy exercises for AI-driven assessment and monitoring

**DOI:** 10.1016/j.dib.2026.112635

**Published:** 2026-02-28

**Authors:** Md. Tauhid Bin Iqbal, Md. Tawhid Mostafa, Md. Tanvir Ahmed, Md. Faiaz Fahim, Md. Sagir Ahmed, Anik Ahamad, Khawja Redwanul Islam, Byungyong Ryu, Gihun Song, Md. Zahid Hossain

**Affiliations:** a Department of Computer Science and Engineering, East West University, Aftabnagar, Dhaka 1212, Bangladesh; b Department of Computer Science and Engineering, Stamford University Bangladesh, Mughda, Dhaka 1214, Bangladesh; c Augmedix, Tejgaon I/A, Dhaka 1208, Bangladesh; d Digital Health, Deggendorf Institute of Technology, 94469 Deggendorf, Germany; e University of Wales Trinity Saint David, Birmingham, 636A, United Kingdom; f Meta Learn Inc., Hwaseong-si- 18469, South Korea; g LG Electronics, AI Lab, Seoul- 06772, South Korea; h Department of Physiotherapy and Rehabilitation, Jashore University of Science and Technology, Jashore 7408, Bangladesh

**Keywords:** 2-dimensional visual dataset, Visual exercise evaluation, Rehabilitation support systems, AI-guided remote physical therapy, Exercise assessment questionnaires

## Abstract

We present *MobiPhysio*, a 2D video-based dataset designed to support AI-driven physiotherapy assessment and monitoring. The dataset contains 3686 segmented videos of 9 Active Range of Motion physiotherapy exercises performed by 58 male and female participants. The recordings are done under the variations in lighting, camera angles, occlusion, and jitter in order to mimic real-world conditions. Data collection occurred in two phases: first from non-expert participants at Stamford University Bangladesh, and later from expert participants at the Department of Physiotherapy and Rehabilitation, Jashore University of Science and Technology. The entire process was conducted under the guidance of certified physiotherapists. Each video is further annotated with assessment scores derived from the exercise-specific Exercise Accuracy Assessment Questionnaire (EAAQ), developed under expert guidance. This dataset would enable researchers to build and test AI-powered physiotherapy and rehabilitation systems, examine human motion, and create exercise monitoring solutions using available 2D camera devices like mobile phones without the need of external body-reliant sensors.

Specifications Table

**Table:** 

Subject	Computer Sciences
Specific subject area	AI and Computer Vision for Physiotherapy Exercise Assessment
Type of data	Video Raw, Analyzed
Data collection	We collected exercise videos from expert and non-expert participants. Eight mobile devices were used to record the exercise videos: Vivo Y20G, Samsung Galaxy S20 FE 5 G, Redmi 6A, Vivo Y11, Huawei Y541-U02, OnePlus 9, OnePlus 8T, and Pixel 6A. The recordings were made with a 380A tripod and were kept at about 1100 mm height throughout the data collection process.
Data source location	The data were collected from the following two locations. 1. Stamford University Bangladesh, Dhaka, Bangladesh (Latitude: 23.7394° N, Longitude: 90.3843° E) 2. Jashore University of Science & Technology, Jashore, Bangladesh (Latitude: 23.1667° N, Longitude: 89.2167° E).
Data accessibility	Repository name: MobiPhysio Data identification number: 10.7910/DVN/XSI0QN Direct URL to data: https://doi.org/10.7910/DVN/XSI0QN
Related research article	K. R. Islam, M. S. Ahmed, A. Ahamad, K. Fatima, T. Akter, B. Ryu, and M. T. B. Iqbal, “A video-based physiotherapy exercise dataset,” 2022 25th International Conference on Computer and Information Technology (ICCIT), pp. 780–784, 2022. [Online]. Available: https://ieeexplore.ieee.org/document/10055189 [1].

## 1 Value of the Data

• This dataset represents one of the first large-scale collections of 2D video-based physiotherapy exercises recorded under real-world conditions using smartphones.
• Researchers can leverage this dataset to train and benchmark AI models for various tasks such as exercise classification & assessment, pose estimation, movement quality assessment, rehabilitation progress monitoring, and other relevant tasks.
• All recordings were captured using standard 2D smartphones, making the dataset ideal for developing AI models compatible with low-cost 2D-camera devices. This can help promote accessible, at-home rehabilitation solutions for people across various regions and socioeconomic backgrounds who can hardly afford the costly 3D-sensor-based solutions available.
• With videos from both expert and non-expert participants, the dataset enables AI models to understand physiotherapy exercises across varying skill levels and spot common movement errors, providing precise AI monitoring to help individuals perform exercises effectively at home.

## 2 Background

Many people, especially older adults, injured individuals, and those in rural areas struggle to access regular physiotherapy due to distance, limited availability of therapists, and cost. These difficulties became even more serious during epidemics like COVID-19, particularly in rural regions of underdeveloped countries. Private physiotherapy is often costly, so AI-based tools offer a practical way to perform guided exercises at home . Existing systems, including Social Assistive Robots (SAR) [2], KiReS [3], and Exergames [4], largely rely on sensors such as Kinect [7] or wearable devices, and their models are trained using datasets like KIMORE [8], UI-PRMD [9], and IRDS [10]. However, these sensor-based solutions are expensive, complex, and mostly unavailable in many regions. A more accessible approach is to use 2D camera inputs from everyday devices such as smartphones or webcams. While early work [1] showed promise, it was limited by narrow exercise variety, restricted participant demographics, inadequate handling of occlusions, and insufficient expert-reviewed annotations. Similar limitations are also present in other existing efforts, including UCO Physical Rehabilitation [5] and REHAB24–6 [6]. To fill these gaps, we introduce *MobiPhysio*, a 2D video dataset with expert and non-expert recordings, diverse exercises, and reliable labels for AI-driven physiotherapy and rehabilitation research.

## 3 Data Description

**Fig. 1:**
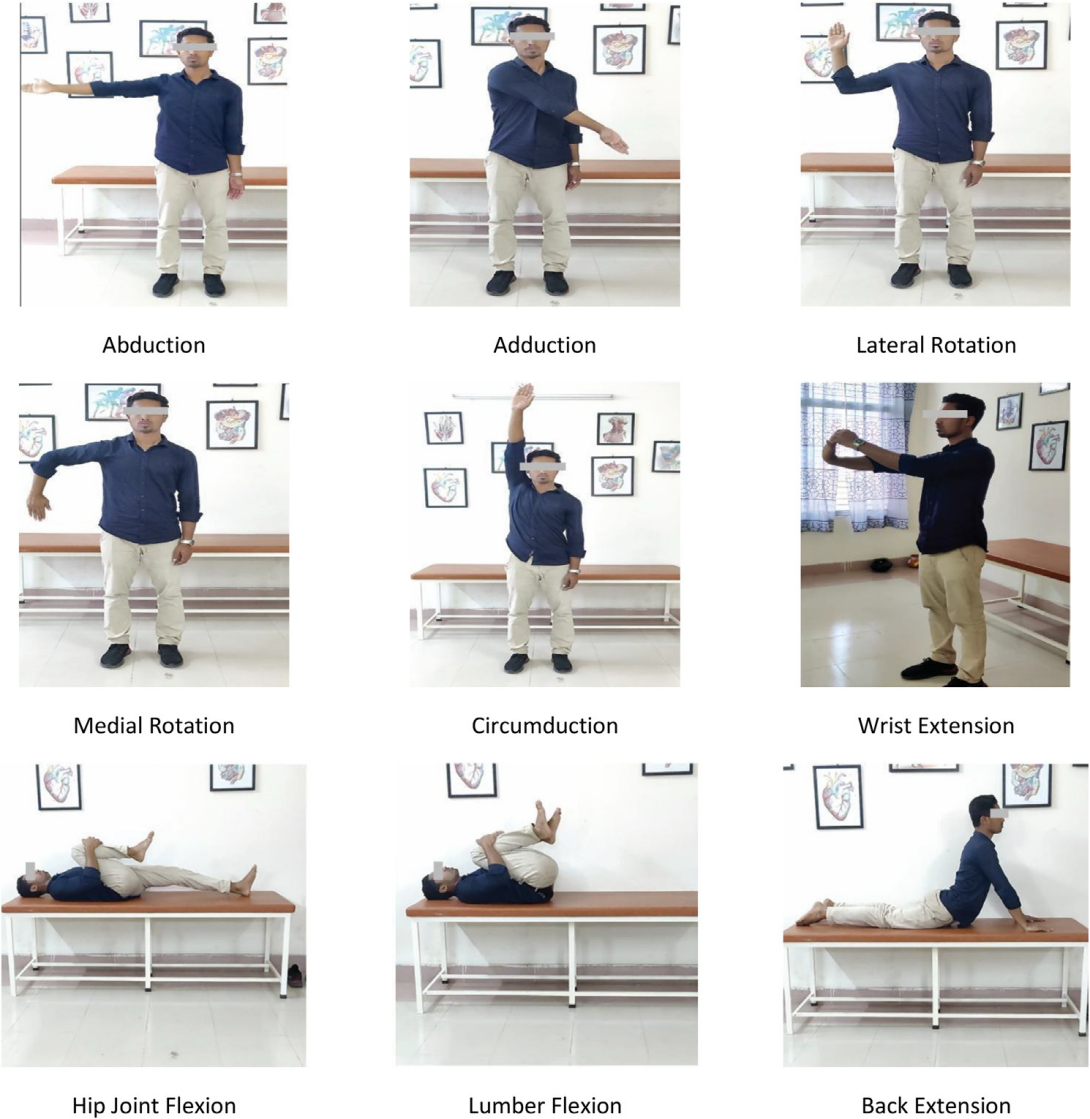
Sample video frames illustrating Physiotherapy Exercises.

Our dataset contains 3686 videos of 9 Active Range of Motion [11] exercises that can be performed independently. These exercises are Abduction (E01), Adduction (E02), Lateral Rotation (E03), Medial Rotation(E04), Circumduction(E05), Wrist Extension (E06), Hip Joint Flexion (E07), Lumber Flexion (E08), and Back Extension (E09), as shown in Fig. 1.

**Fig. 2:**
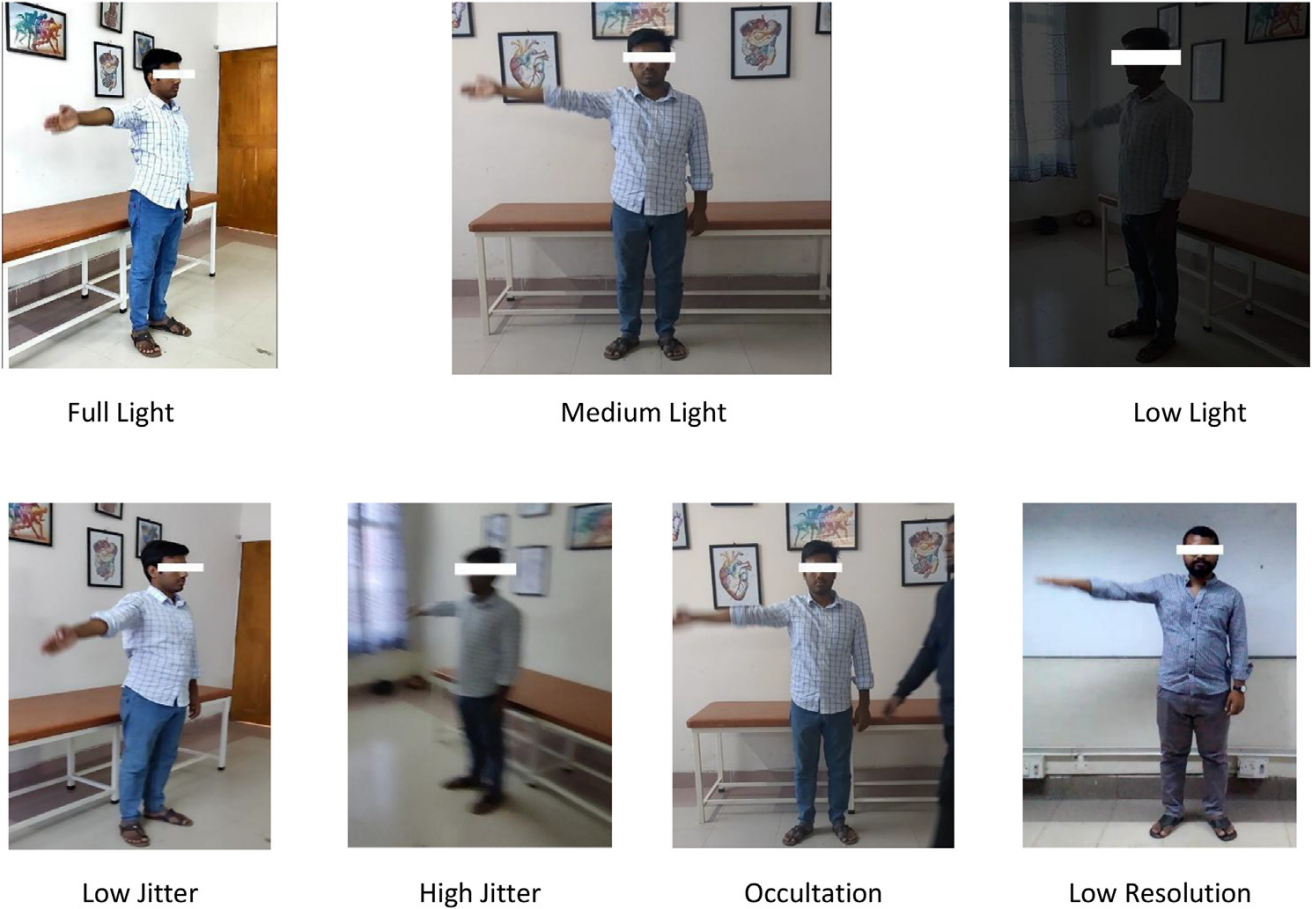
Sample video frames illustrating dataset variations.

The dataset was created in a controlled environment using smartphone cameras under the supervision of certified physiotherapists, with the inclusion of both expert and non-expert participants. Different variations are included in the dataset to mimic the real condition, including Full Light (FL), Medium Light (ML), Low Light (LL), Low Jitter (LJ), High Jitter (HJ), Occultation (O), and Low Resolution (LR). Fig. 2 shows a snapshot of such variations. Variations make the dataset more robust and practical for real-world use. The exercises were recorded in 1080p resolution at 30 frames per second from three angles—front, left, and right, to provide a comprehensive biomechanical analysis opportunity. These exercises help manage conditions such as Frozen shoulder [12], Hip Joint Problem [13], carpal tunnel syndrome [14], Lumbar Spondylosis [15,16], and Low Back Pain [17]. A summary of the exercises is provided in Table 1.

**Table 1:** Dataset Summary.

Problem Types	Exercise Name	Male	Female	Total Person	Expert Group Videos	Non-expert Group Videos	Total Videos
Frozen Shoulder	Abduction	32	19	51	382	176	558
	Adduction	16	8	24	384	0	384
	Circumduction	28	17	45	371	140	511
	Lateral Rotation	32	19	51	383	167	550
	Medial Rotation	16	8	24	383	0	383
Carpal Tunnel Syndrome	Wrist Extension	14	8	22	354	0	354
Low Back Pain, Lumbar Spondylosis	Lumber Flexion	30	0	30	252	104	356
	Back Extension	27	0	27	251	88	339
Adhesive Capsulitis of Hip Joint, Hip Joint Problem	Hip Joint Flexion	14	0	14	251	0	251

All video files are uploaded one by one in the repository [18] without using any folders or subfolders, as per the requirements of the repository. Each file name follows a fixed pattern that includes important details like the exercise type, person ID, camera angle, recording condition, and gender. The structure of the file names is shown in Table 2. Please note that faces in Figs. 1 and 2 are partially masked in line with standard practices to protect participant identity in paper. However, the publicly released dataset [18] contains the original videos with fully visible faces.

**Table 2:** Naming Convention of the exercise videos.

E01	P01	A F	V FL	G M
Exercise Number	Participant Number	Angle (*F* = Front, *L* = Left, *R* = Right)	Variation (FL / ML / LL / LJ / HJ / O / LR)	Gender (*M* = Male, *F* = Female)

## 4 Experimental Design, Materials and Methods

**Fig. 3:**
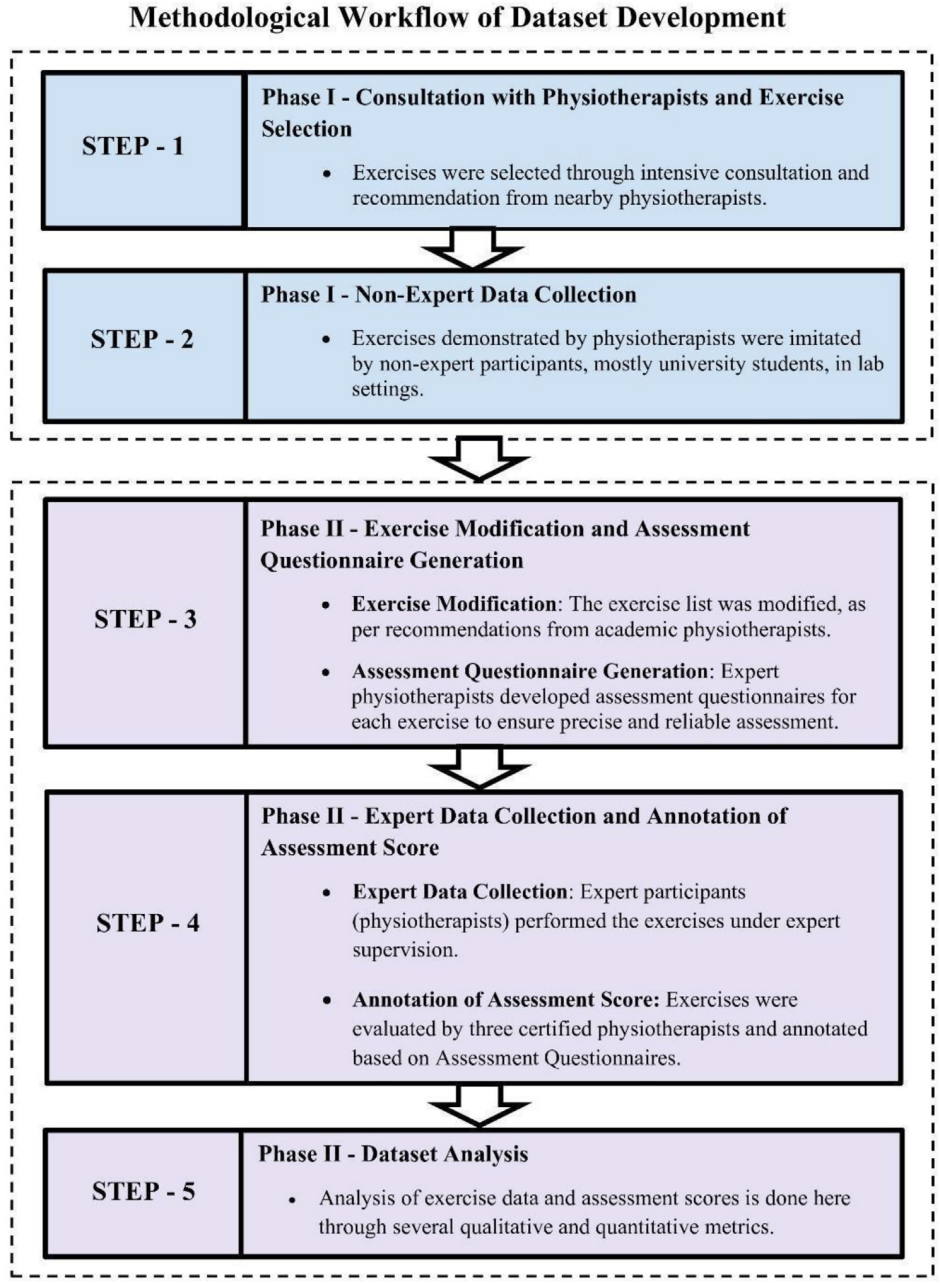
Methodological Workflow of Dataset Development.

This study follows a structured methodology for developing an extensive 2D physiotherapy exercise dataset. The process involved domain-expert consultation, exercise elicitation, and multi-phase video collection from both non-expert and expert participants. Special care was taken to maintain video quality, angle consistency, and proper evaluation protocols. The entire process was guided and validated by certified physiotherapists to ensure clinical relevance. Fig. 3 presents the methodological workflow of the study.

### 4.1 Consultation with physiotherapists and exercise selection

We began the process by consulting physiotherapists experienced in rehabilitation to identify common, manageable conditions suitable for home-based physiotherapy. In phase I, we selected 14 exercises targeting six prevalent issues—frozen shoulder, low back pain, carpal tunnel syndrome, neck pain, knee pain, and tennis elbow. These exercises were recommended for their high incidence across age groups and the need for regularity to ensure proper recovery. The selected exercises could be performed at home without assistance using the available 2D cameras of smartphones or laptops. To ensure accurate references, physiotherapists provided model videos performed by themselves that demonstrate the proper way of doing the exercises.

### 4.2 Non-Expert data collection

In phase I, we recorded exercise videos from individuals without any formal expert supervision. Our goal was to reflect a realistic home-based physiotherapy scenario. Exercises were carried out by participants who imitated the model exercise videos (performed by the physiotherapists) in a controlled lab environment. This helped ensure that the dataset captures the kind of variations and challenges typically faced by actual patients performing exercises at home. At this stage, we recorded a total of 1237 exercise videos from 28 healthy volunteers, comprising 25 males and 3 females, all aged between 18 and 28. All participants were students from the Department of Computer Science and Engineering at Stamford University Bangladesh. They volunteered to take part in the study and had no prior background or training in physiotherapy, making them ideal candidates to represent typical non-expert users. All participants performed the exercises voluntarily, following demonstration videos provided earlier.

To ensure realistic and diverse video samples, the videos were recorded using five commonly available mobile phone models—Vivo Y20G, Samsung Galaxy S20FE, Redmi 6A, Vivo Y11, and Huawei Y541-U02—mounted on a 380A tripod. Videos were captured from multiple angles (front, left-side, and right-side) and variations such as lighting conditions (low light and extreme low light), low jitter, high jitter, and low resolution. They are used in recordings to mimic potential real-world scenarios. Each exercise class was performed by participants and recorded with eight different environmental variations. The details were reported in our earlier work [1].

## 5 Exercise Modification and Assessment Questionnaire Generation

### 5.1 Exercise modification

Following up on our prior work [1], we tried to enhance our data collection. To ensure the data was clinically reliable and practically useful, we sought guidance from certified and experienced physiotherapists. For this reason, we collaborated with the Department of Physiotherapy and Rehabilitation at Jashore University of Science and Technology (JUST), which at that time was the only department in a public university of Bangladesh for academic education and research in physiotherapy. This collaboration was the beginning of the data modification phase, where the initial set of exercises was critically reviewed and carefully narrowed down into 9 Active Range of Motion [11] exercises. The modifications were shaped by three key considerations:

1. Some of the earlier exercises were closer to fitness routines or required assistance (Passive Range of Motion [11]), which made them less practical for home use. The revised set emphasizes Active Range of Motion [11] exercises that patients can perform on their own.
2. The experts suggested enriching the collection with more demonstrations from trained physiotherapists and balancing male and female participation, making the dataset more inclusive and broader.
3. They also recommended incorporating occlusion instances where body parts are partially blocked, to better reflect real-world home environments and strengthen model robustness.

This refinement was carried out under the guidance of Dr. Md. Zahid Hossain (PT), Chairman, and Dr. Farzana Sharmin Liza (PT), Department of Physiotherapy and Rehabilitation, Jashore University of Science and Technology.

### 5.2 Assessment Questionnaire Generation

We aim to develop an AI-monitored home-based physiotherapy assessment system using the collected dataset. The primary goal of this system is not only to classify the type of exercise performed at home but also to evaluate its quality through meaningful assessment scores. Such feedback will help patients improve their prescribed exercises and support effective recovery. To achieve this, reliable scoring for each video is essential. According to our expert physiotherapists, exercises differ greatly in form and purpose. Therefore, they recommended the use of exercise-specific questionnaires to ensure more accurate and clinically relevant scoring.

Accordingly, we developed the Exercise Accuracy Assessment Questionnaire (EAAQ) for each exercise, focusing on posture, movement accuracy, and adherence to clinical standards. The process began with input from Dr. Humayun Kabir (PT) from Gonoshasthaya Samaj Vittik Medical College Hospital, followed by refinement under the guidance of Dr. Md. Zahid Hossain (PT), Dr. Farzana Sharmin Liza (PT), and their team at JUST.

The questionnaire includes seven carefully designed questions, grouped into two main categories:

**Primary Factors (PF):** The first four questions focus on the basics, whether the person is performing the exercise at the correct speed and completing the recommended number of repetitions.

**Control Factors (CF):** The remaining three questions are designed to evaluate posture and movement quality.

**Fig. 4:**
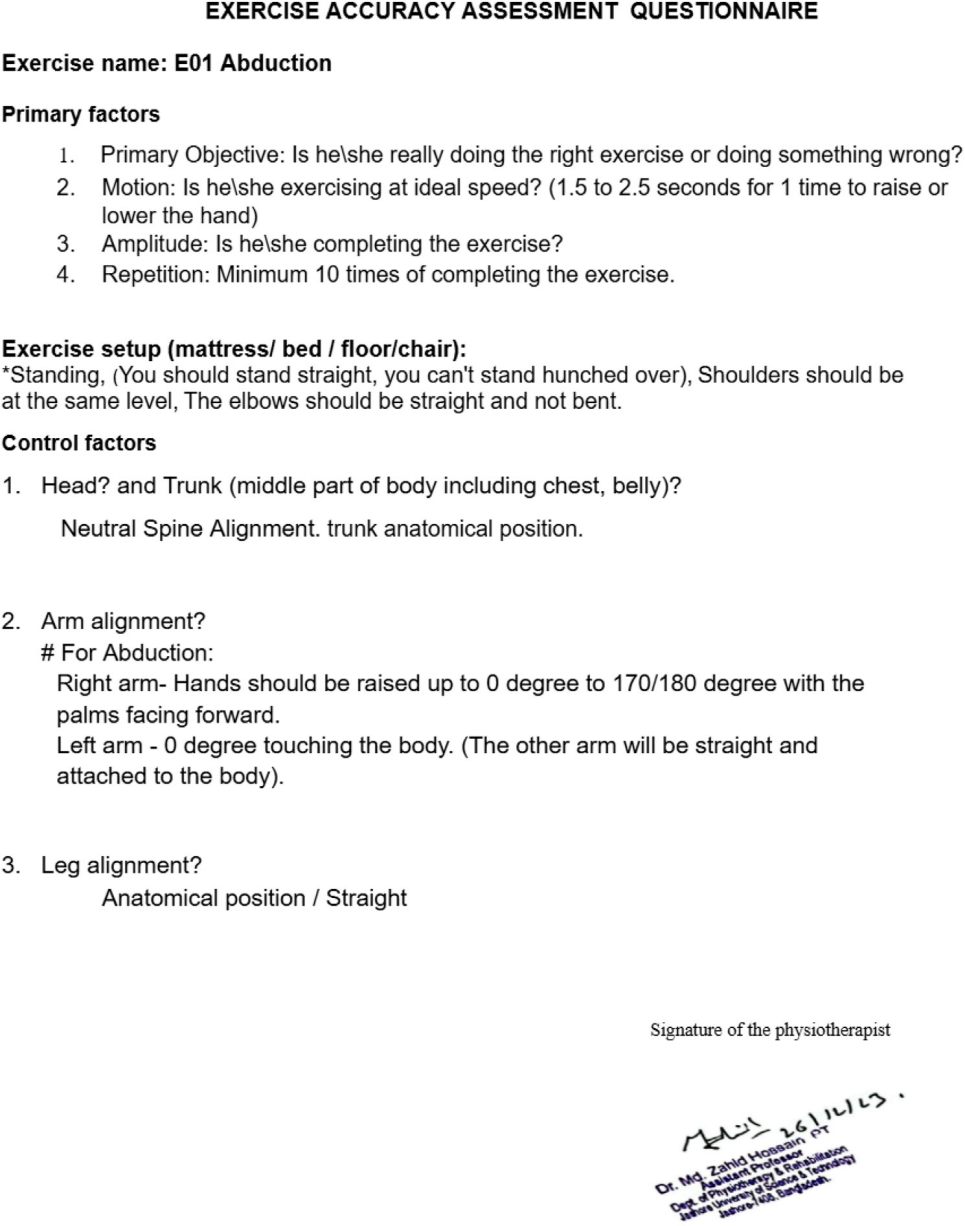
Exercise Accuracy Assessment Questionnaire for Abduction.

A point-wise distinction between phase I and phase II is given in Table 3. One sample questionary is provided at Fig. 4, and the others are provided in the Appendix.

**Table 3:** Distinction between Phase I and Phase II Data Collection.

Aspect	Phase I	Phase II
Exercise Selection	14 exercises selected by practicing physiotherapists	Meticulously narrowed down to 9 physiotherapy exercises based on expert recommendation
Number of Videos	1237 videos	3686 videos
Involvement of Expert/ Non-expert Participant	1237 videos collected from non-expert participants	3011 videos from expert and 675 videos from non-expert participants
Variations	Front View, Left View, Right View, Low Jitter, High Jitter, Low Light, Extreme Low Light, Low Resolution	Full Light, Medium Light, Low Light, Low Jitter, High Jitter, Occultation, and Low Resolution; each recorded from three angles: Front, Left, and Right
Gender Diversity	Very limited female participant (3 Females)	Enhanced the female participant videos significantly (19 Females)
Use of Exercise Assessment Questionnaire	Generic questionnaire for all exercise	Exercise-specific questionnaire
Involvement of domain experts in labeling	Labelling partially done by domain-experts	Labeling fully done by three domain-experts

## 6 Data Collection and Annotation of Assessment Score

### 6.1 Expert data collection

After finalizing the exercise list and developing exercise-specific evaluation questionnaires, we initiated the expert data collection phase at the Department of Physiotherapy and Rehabilitation, Jashore University of Science and Technology (JUST). The 9 selected exercises were performed under controlled conditions by the students of the Physiotherapy and Rehabilitation department, equipped with both academic knowledge and practical training in physical rehabilitation. To maintain consistency and clarity, we first recorded model demonstration videos for all exercises, performed by Dr. Imrul Kayes (PT) from JUST, and shared them with the participants as reference. A total of 24 expert participants (16 male and 8 female students) contributed to the recordings, ensuring accurate execution of each exercise.

Altogether, 3011 videos were collected. Each exercise was captured under varied conditions, including changes in lighting (full, low, and extreme low light), motion stability (high and low jitter), and occlusion. Every variation was further recorded from three angles (front, left, right) using three different mobile devices (OnePlus 9, OnePlus 8T, Google Pixel 6A). All recordings were made in 1080p at 30 fps, providing high-quality footage for motion analysis. To standardize the setup, a 380A tripod at a consistent height of 1100 mm was used throughout. These expert participants ensured that each exercise was performed in precise form and technique, while the inclusion of minor variations reflected realistic user performance patterns.

### 6.2 Annotation of assessment score

All recorded exercises were evaluated using the EAAQs by three certified physiotherapists—Dr. Mamun Mahmud (PT), Dr. Abid Hasan Khan (PT), and Dr. Rayhan Islam (PT), from JUST. Each physiotherapist independently assessed the videos, focusing on key aspects such as form, technique, range of motion, and adherence to prescribed movements. Their scores were then averaged to generate the final score. For better clarity and usability, the averaged scores were scaled to a 100-point system, making the results easier to interpret for both model development and performance analysis. Finally, we measured the level of agreement among annotators using multiple metrics, including correlation analysis, the Intraclass Correlation Coefficient (ICC) [19], and the Mann-Whitney U test [20].

**Fig. 5:**
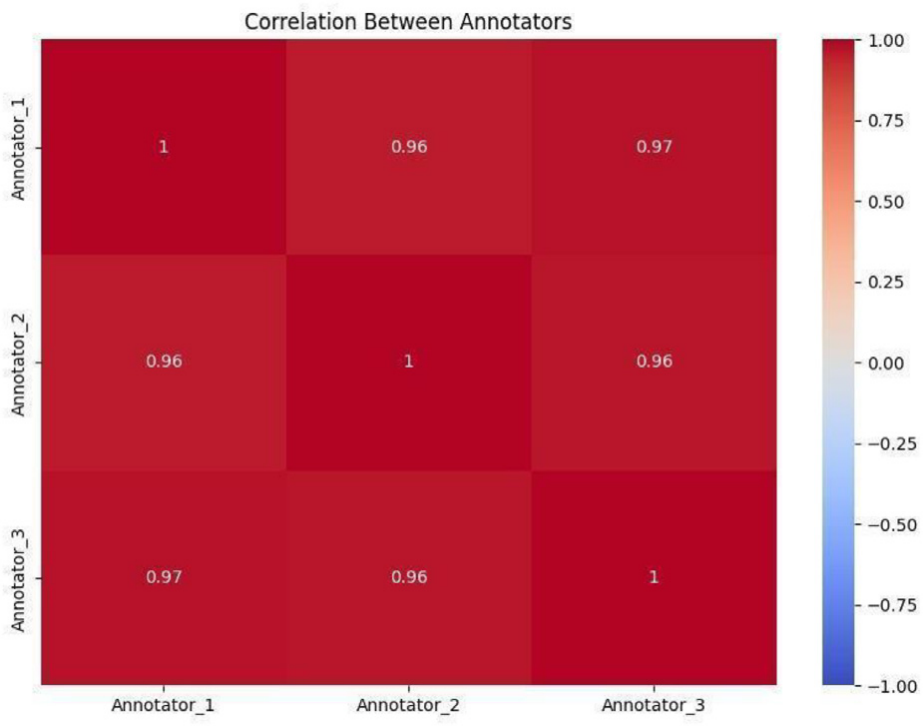
Heatmap of annotator scores with strong correlations (0.96–0.97).

1. **Correlation Analysis:** We observed strong pairwise correlations between annotators’ scores, ranging from 0.96 to 0.97, as shown in the heatmap (Fig. 5). This high level of agreement suggests that the annotators followed similar grading patterns. It reflects a consistent and shared understanding of the performed exercises. This consistency is further supported by the violin plots (Fig. 6), which show that most ratings from all three annotators fall between 5 and 7. While the plots reveal some subtle variations in score distributions indicated by slight differences in the widths of the violins, these are minor and suggest that each annotator applied the scoring system with steady but slightly individual interpretation. Overall, the results demonstrate strong reliability in the evaluation process.

**Fig. 6:**
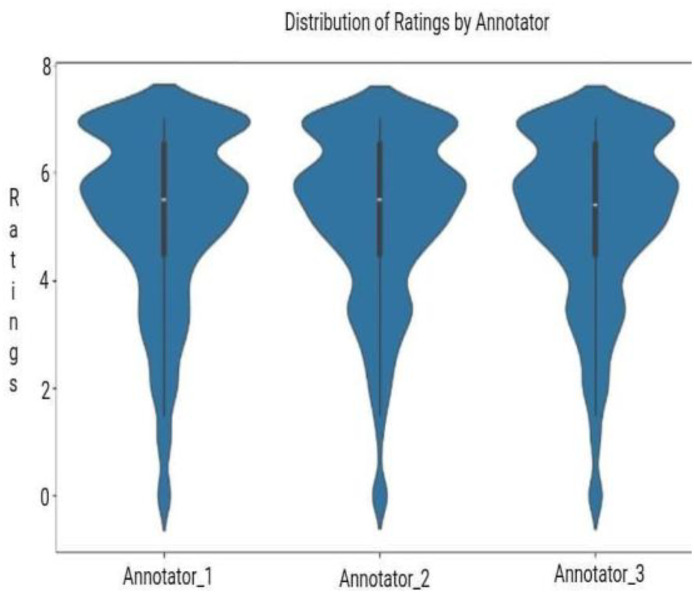
Violin plots of annotator ratings (5–7 range).
2. **Intraclass Correlation Coefficient (ICC(3,k)):** Given that the ratings are collected in fractional values, we use the Intraclass Correlation Coefficient (ICC) to measure the reliability among annotators. The ICC(3,k) value of 0.6672 indicates a moderate to good level of agreement across the averaged scores. This reflects a strong overall similarity in how annotators evaluated the exercises, though some variation remains likely due to individual differences in perception and judgment.
3. **Mann-Whitney U Test:** To further explore how closely the annotators agreed, we used the Mann-Whitney U test to check for any significant differences in their scoring distributions. The results showed that all p-values were above 0.05, indicating no statistically significant differences between any pair of annotators (Annotator 1 vs. Annotator 2: *p* = 0.1350, Annotator 1 vs. Annotator 3: *p* = 0.1533, Annotator 2 vs. Annotator 3: *p* = 0.9269). These findings support the idea of consistent scoring across annotators. The strong correlation values (ranging from 0.958 to 0.971) and the low mean absolute differences (between 0.208 and 0.282) further confirm this consistency, suggesting that while annotators may differ slightly, their overall scoring patterns are very much in sync.

Note that a CSV file containing expert assessment scores for all videos, along with a README file summarizing exercises and participant-level information, is provided in [18].

### 6.3 Dataset analysis

To provide an overview of the dataset, we present a set of analytical visualizations that highlight the quality and distribution of physiotherapy exercises.

**Fig. 7:**
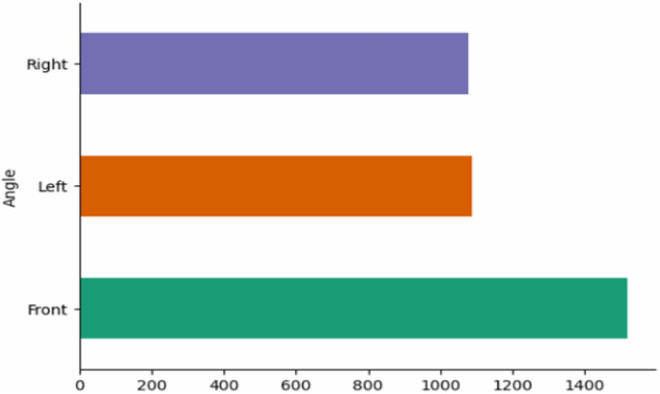
Distribution of Videos Based on Camera Angles.

**Fig. 8:**
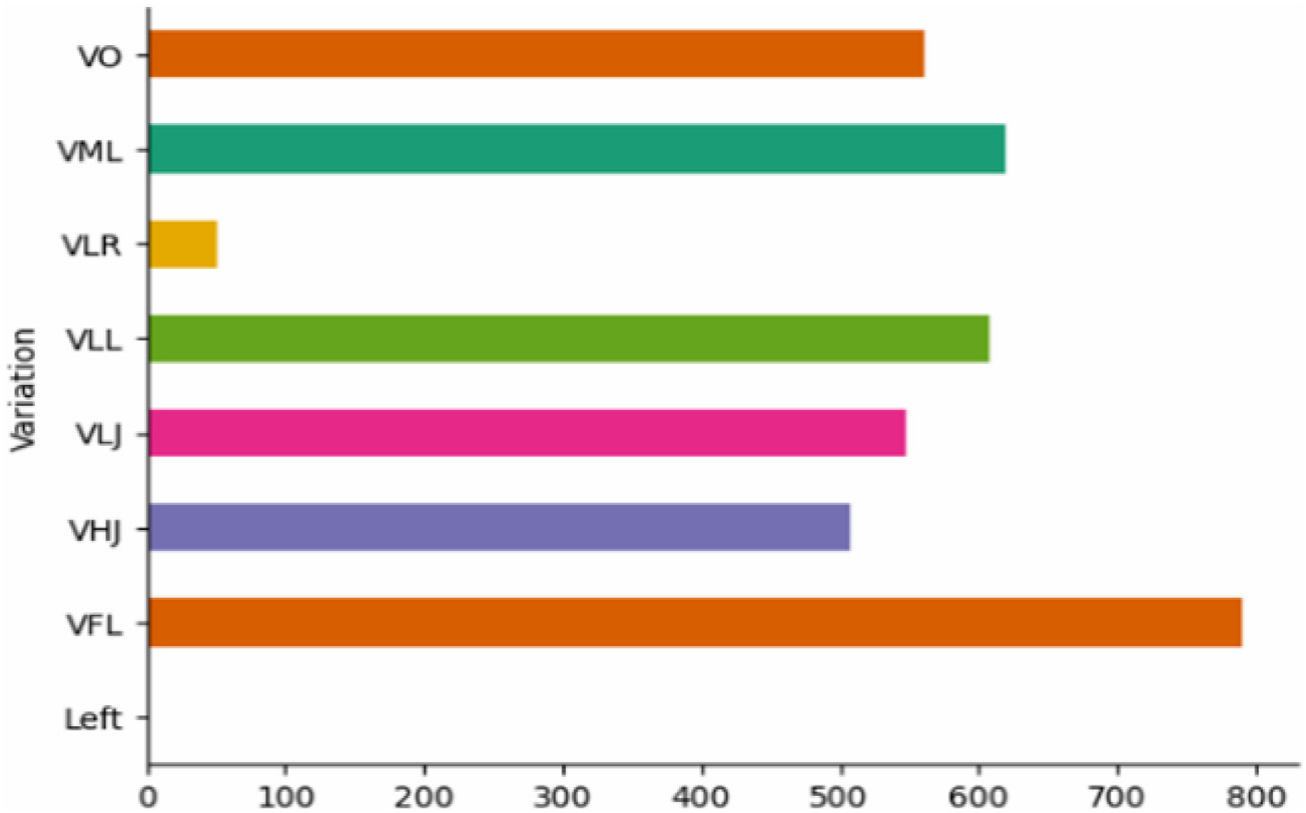
Distribution of Videos Based on Video Variations.

**Fig. 9:**
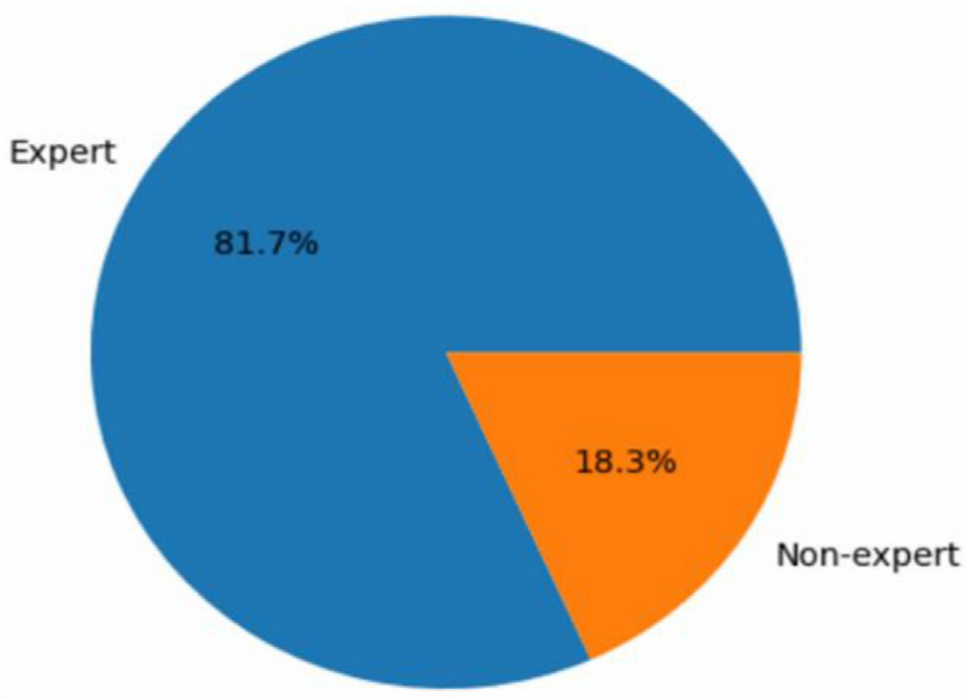
Distribution of Videos Based on Source.

Figs. 7, 8, and 9 provide an overview of the dataset’s composition, variations, and participant contributions. As shown in Fig. 7, videos were recorded from three angles—front, left, and right—with the front view being the most common (1450 recordings), about 32 % more than the side views (1100 each). The front angle provides a clear overall perspective for evaluating posture and movement, while the side angles capture finer details such as joint motion and body alignment.

Fig. 8 summarizes the distribution of diverse real-world variations, including lighting, occlusion, jitter, and resolution. Although low-resolution videos are fewer, the other categories are well balanced. This combination of multiple perspectives and realistic conditions improves the model’s ability to assess exercises accurately and ensures robust performance across different home environments.

Fig. 9 shows that expert participants contributed the majority of videos (81.7 %), with non-experts providing the remaining 18.3 %. Expert recordings offer precise examples of physiotherapy exercises, helping the AI model learn proper form and technique, while non-expert videos introduce natural variability, enhancing the model’s ability to handle less consistent inputs in real-world scenarios.

**Fig. 10:**
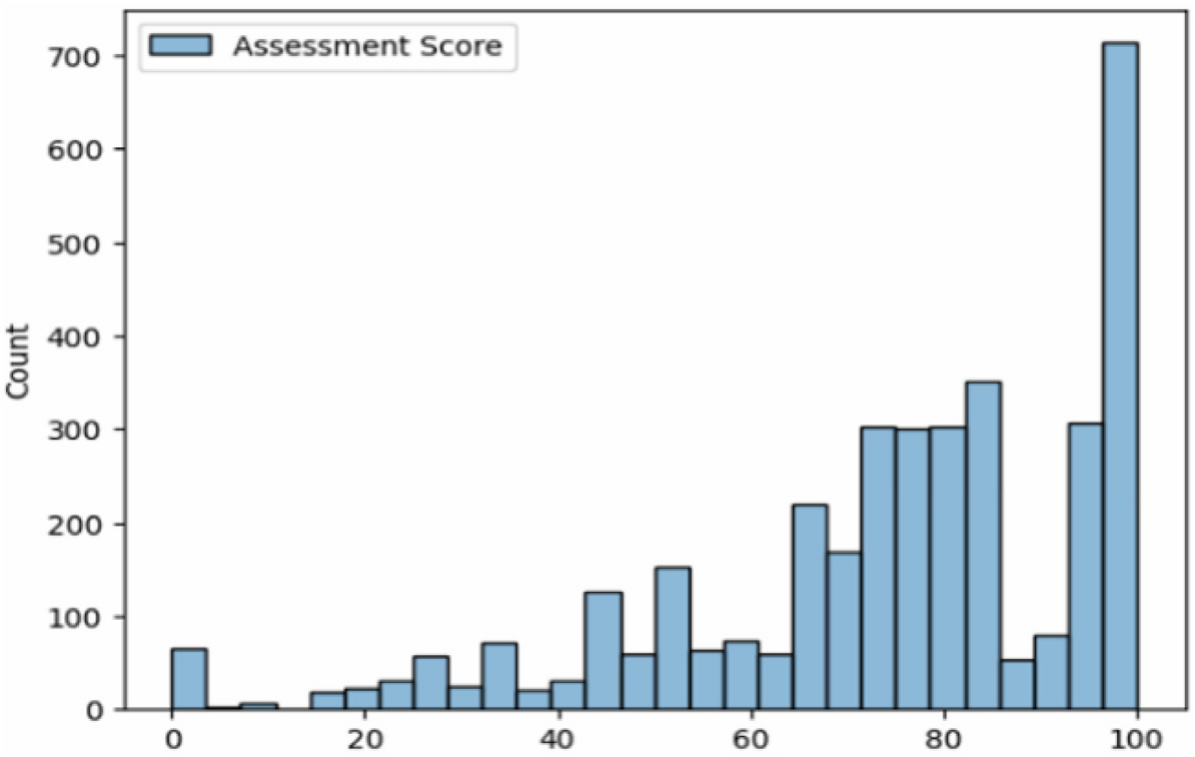
Histoplot of Assessment Score.

**Fig. 11:**
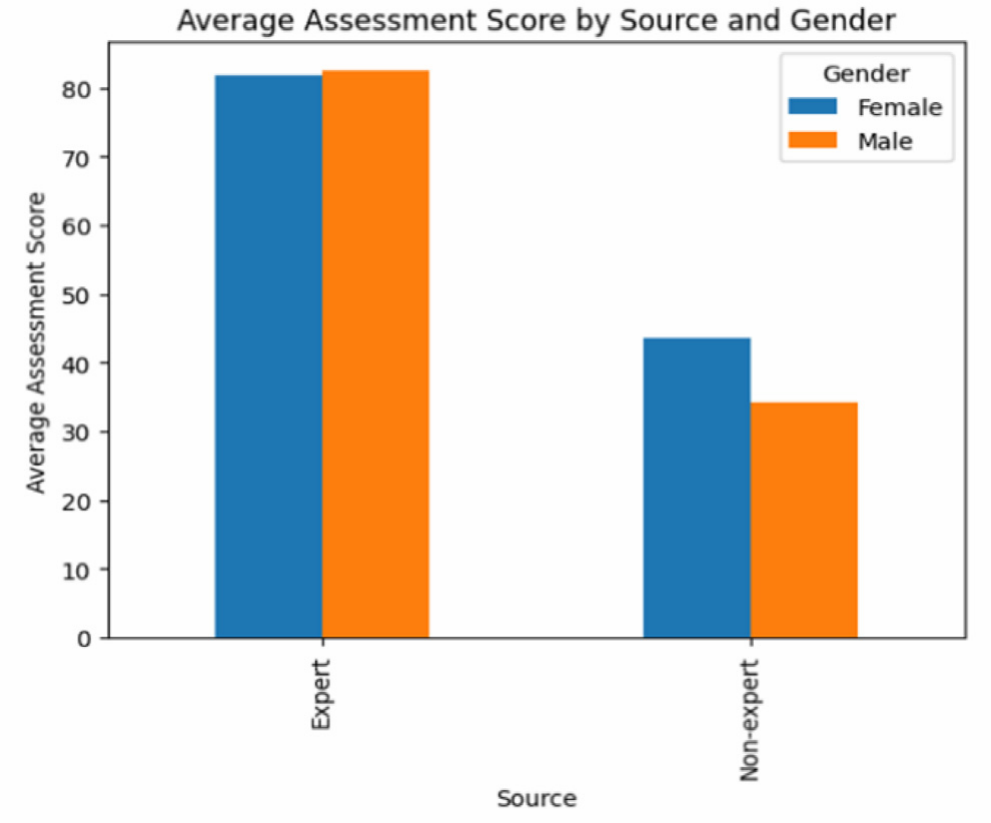
Distribution of Average Assessment Score Based on Source and Gender.

Figs. 10 and 11 illustrate the distribution and comparison of assessment accuracy scores across the dataset. As shown in Fig. 10, exercise scores range from 0 to 100, with many exercises clustered at 100, reflecting high-precision performance under controlled conditions. Scores between 40 and 80 capture natural variability, particularly among non-expert participants or for inherently challenging exercises. However, the dominance of high scores is due to most recordings being performed by physiotherapy-trained experts. While lower-scored exercises are fewer, the dataset still provides substantial variation to support pilot AI training and assessment. We acknowledge that adding more under-performed examples would further enhance model robustness and generalization, which we consider one of our potential future endeavours.

Fig. 11 further breaks down the average scores by user expertise and gender. As observed, experts achieved consistently higher results, averaging 83.2 for males and 82.1 for females, whereas non-experts scored significantly lower, with averages of 35.4 and 44.7, respectively. This reveals a near twofold performance gap between expert and non-expert groups. Furthermore, while expert scores are relatively balanced across genders, non-expert females show a slight advantage over their male counterparts.

## Limitations

We discuss the summary of limitations of the current version of this dataset:

• The dataset primarily includes young, healthy students and physiotherapy experts, as data collection was conducted in an academic setting with participants who were readily available and medically fit. As a result, older adults and individuals with movement impairments are not represented.
• As shown in Fig. 10, the dataset is skewed towards correctly performed exercises, with relatively few poor-quality samples. This imbalance may limit the AI model’s ability to learn from common errors, limiting its generalization in real-world physiotherapy assessment.
• Among the 58 participants, 39 were male and 19 were female. Due to sociocultural norms in Bangladesh, many women were hesitant to participate in publicly visible exercises—particularly those involving lying or sitting, leading to a degree of underrepresentation of female participants in certain exercise types.

Future work will strongly focus on expanding participant demographic diversity, incorporating real physiotherapy patients, and collecting more under-performed exercises to broadly represent performance variability. Meanwhile, to partially mitigate current limitations, different augmentation techniques including motion data augmentation like pose-based synthesis or controlled key point perturbation etc. can be used to simulate common execution errors and movement variations.

## Ethics Statement

This study was conducted in full accordance with ethical standards and institutional guidelines. All data used were collected with informed consent from participants, ensuring respect for privacy and voluntary involvement. No animals or data from social media platforms were used. The research involved no deception or coercion, and no conflicts of interest were declared. All procedures followed the regulatory and ethical principles set by the relevant authorities. The research and its associated data collection procedures were reviewed and approved by the Institutional Review Board (IRB) of the Department of Physiotherapy and Rehabilitation, Jashore University of Science and Technology (JUST), under the approval number **PTR-JUST/IRB/2024/03/42**. A copy of the official IRB approval letter has been provided as a supplementary document.

## Author Statement

**Md. Tauhid Bin Iqbal:** Conceptualization, Data curation, Validation, Writing - Review & Editing, Supervision, Funding acquisition, Investigation

**Md. Tawhid Mostafa:** Methodology, Software, Writing - Original Draft, Data Curation, Formal analysis

**Md. Tanvir Ahmed:** Methodology, Software, Writing - Original Draft, Data Curation, Formal analysis

**Md. Faiaz Fahim:** Methodology, Software, Data Curation, Formal analysis

**Md. Sagir Ahmed:** Methodology, Writing - Review & Editing, Data Curation

**Anik Ahamad:** Writing - Review & Editing, Data Curation

**Khawja Redwanul Islam:** Writing - Review & Editing, Data Curation

**Byungyong Ryu:** Validation, Writing - Review & Editing, Funding acquisition

**Gihun Song:** Validation, Writing - Review & Editing, Funding acquisition

**Md. Zahid Hossain:** Data Curation, Supervision, Validation, Investigation.

## Data Availability

DataverseMobiphysio (Original data).

## Appendix Supplementary materials

Image, application 1
