# Supplementary material for: MobiPhysio: A 2D video dataset of physiotherapy exercises for AI-driven assessment and monitoring
Source: Data Brief. 2026 Feb 28;65:112635. doi: 10.1016/j.dib.2026.112635 (PMC12992533; doi:10.1016/j.dib.2026.112635)
Supplement: Supplementary file 1 [file mmc1.docx]

APPENDIX

The screenshots of all the exercise-specific questionnaires are provided in the Appendix.

| 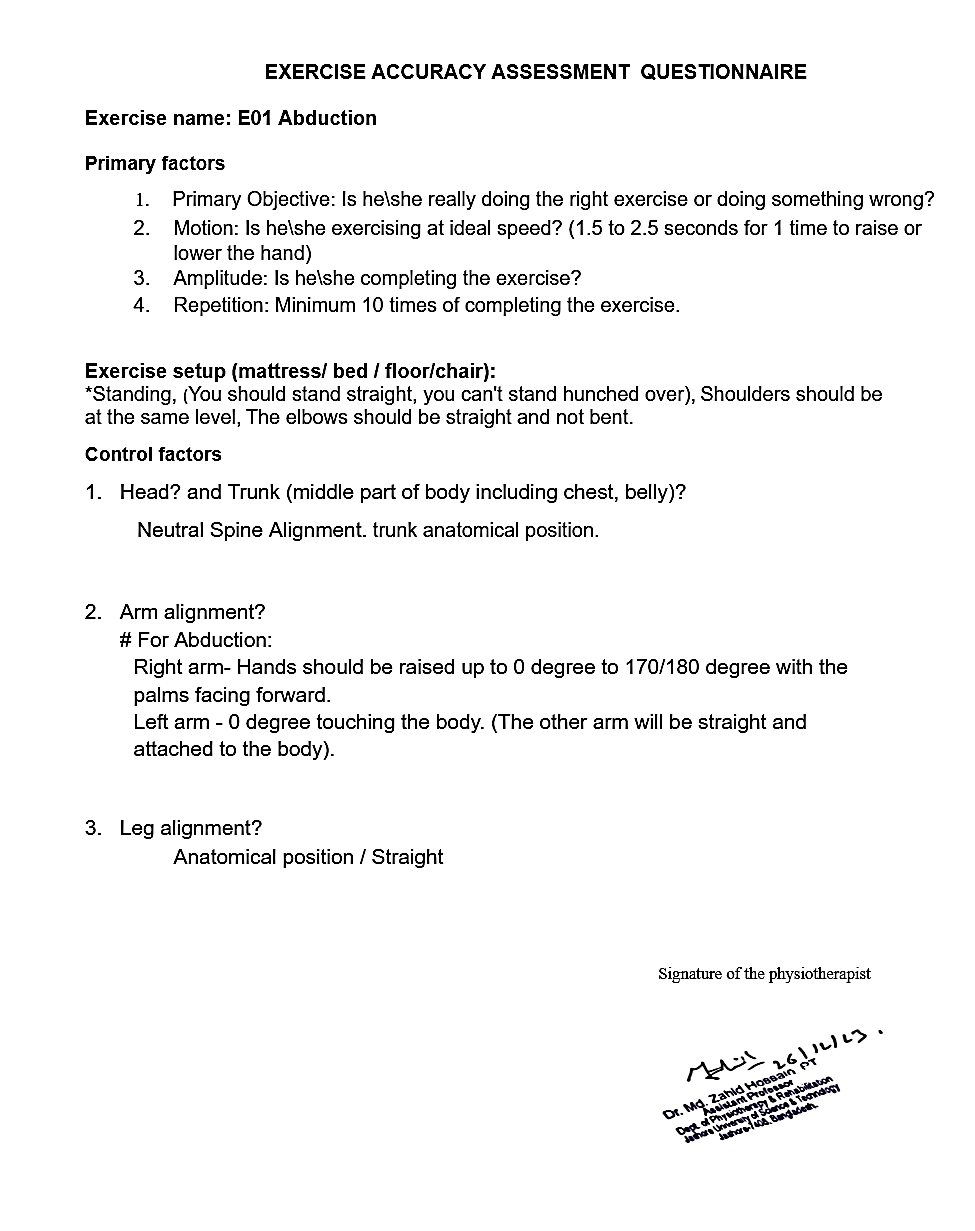  Figure 12.a: Exercise Accuracy Assessment Questionnaire for Abduction |
| --- |
|  |
| 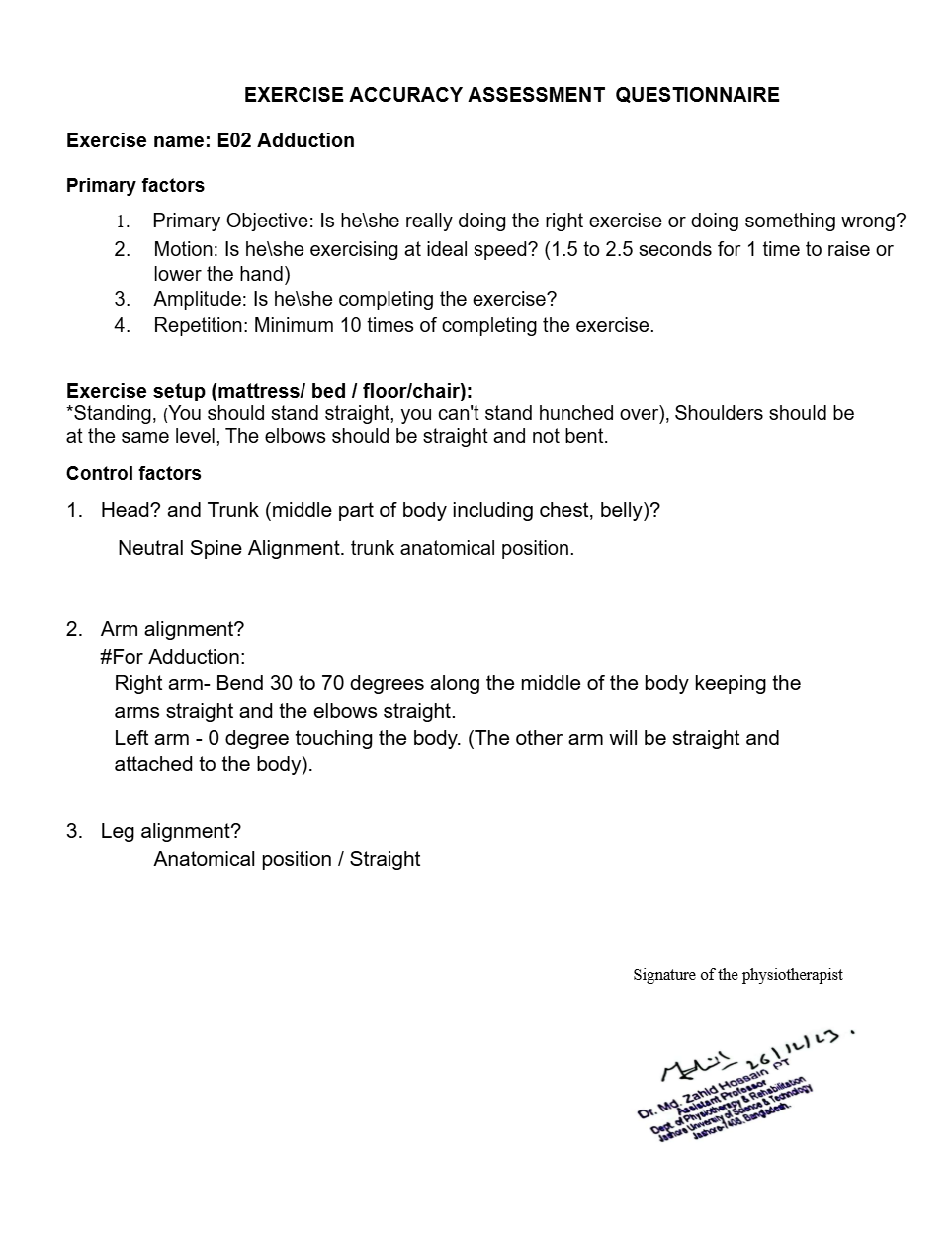 |
| Figure 12.b: Exercise Accuracy Assessment Questionnaire for Adduction |
| 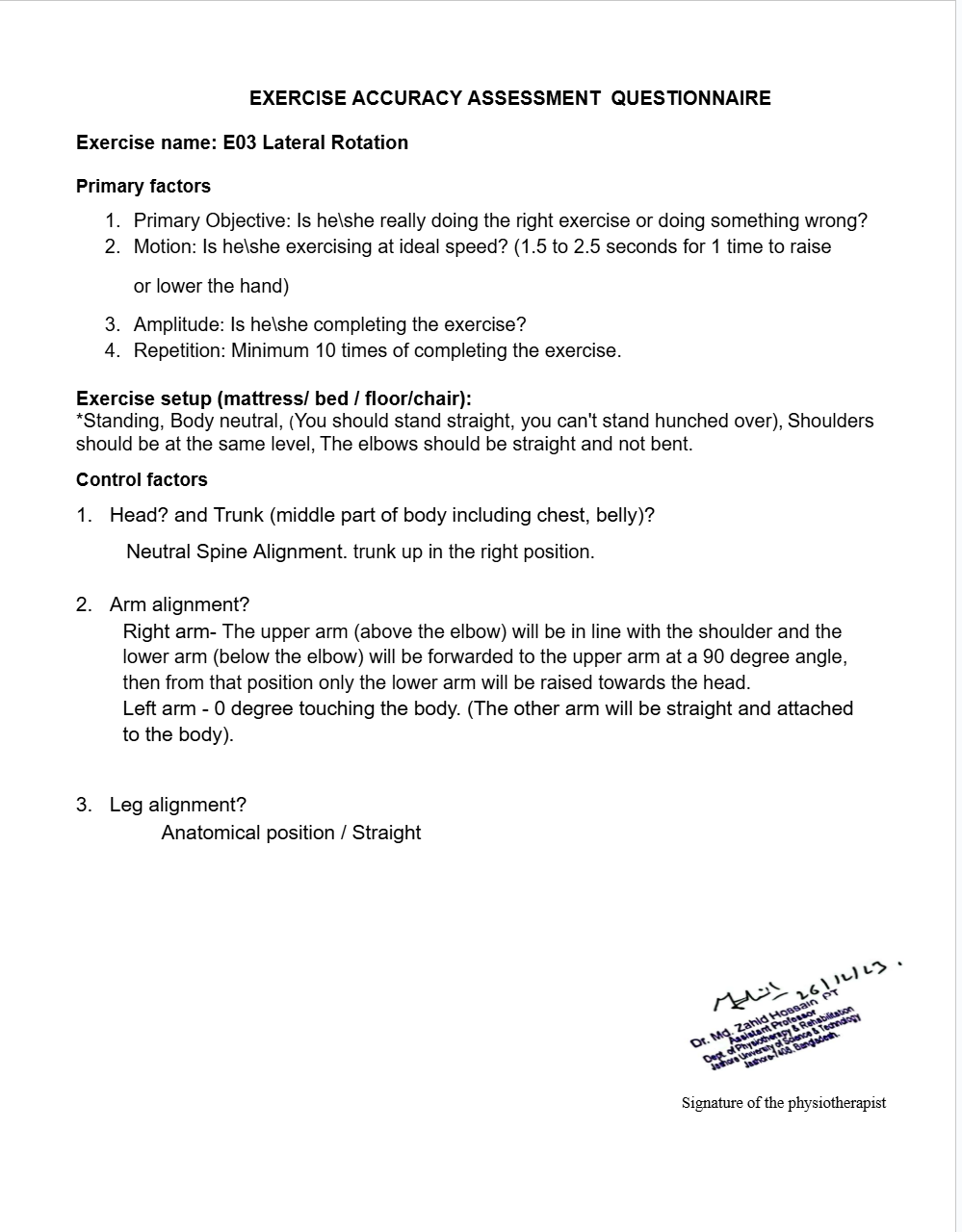 |
| Figure 12.c: Exercise Accuracy Assessment Questionnaire for Lateral Rotation |
| 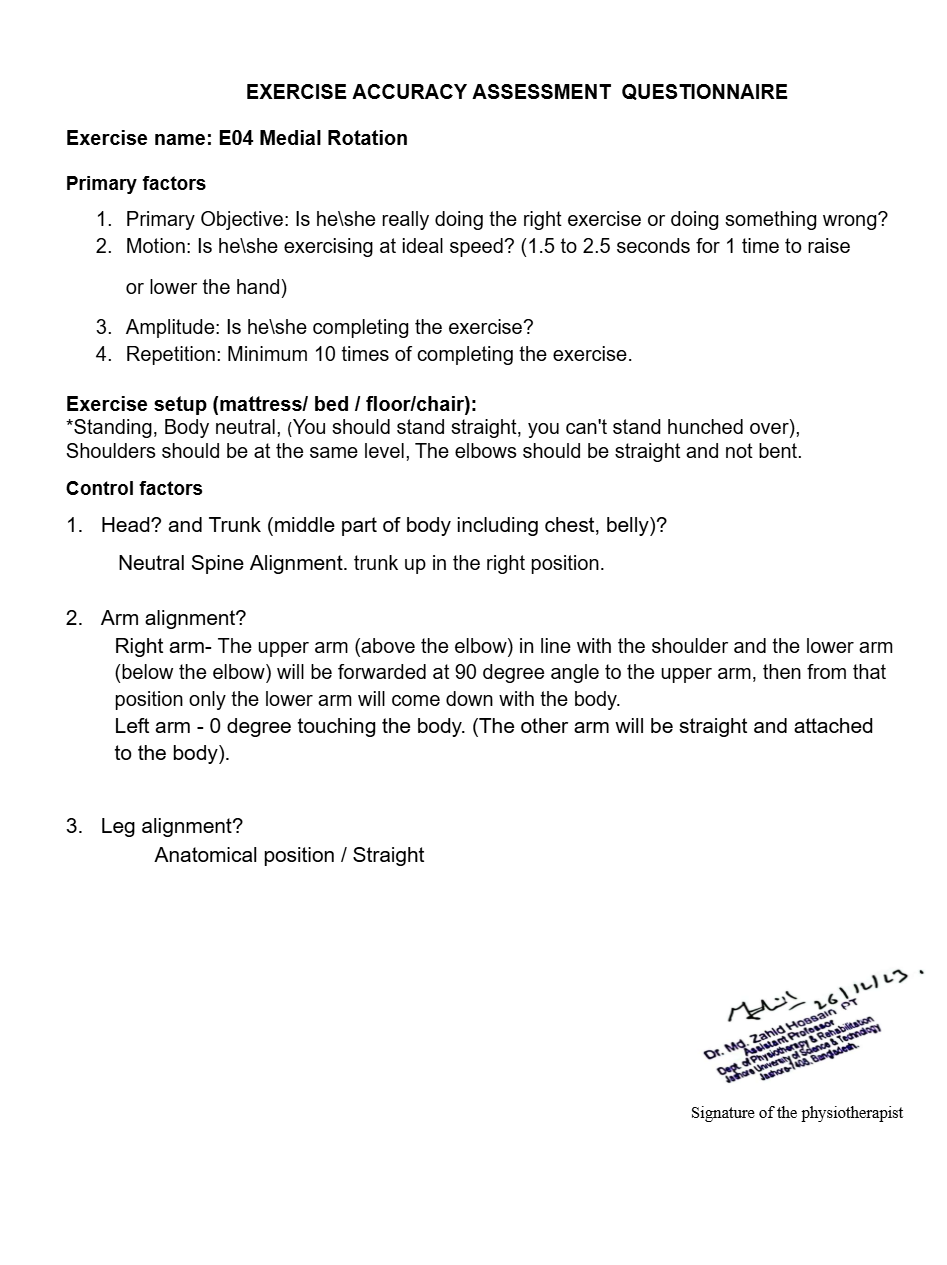 |
| Figure 12.d: Exercise Accuracy Assessment Questionnaire for Medial Rotation |
| 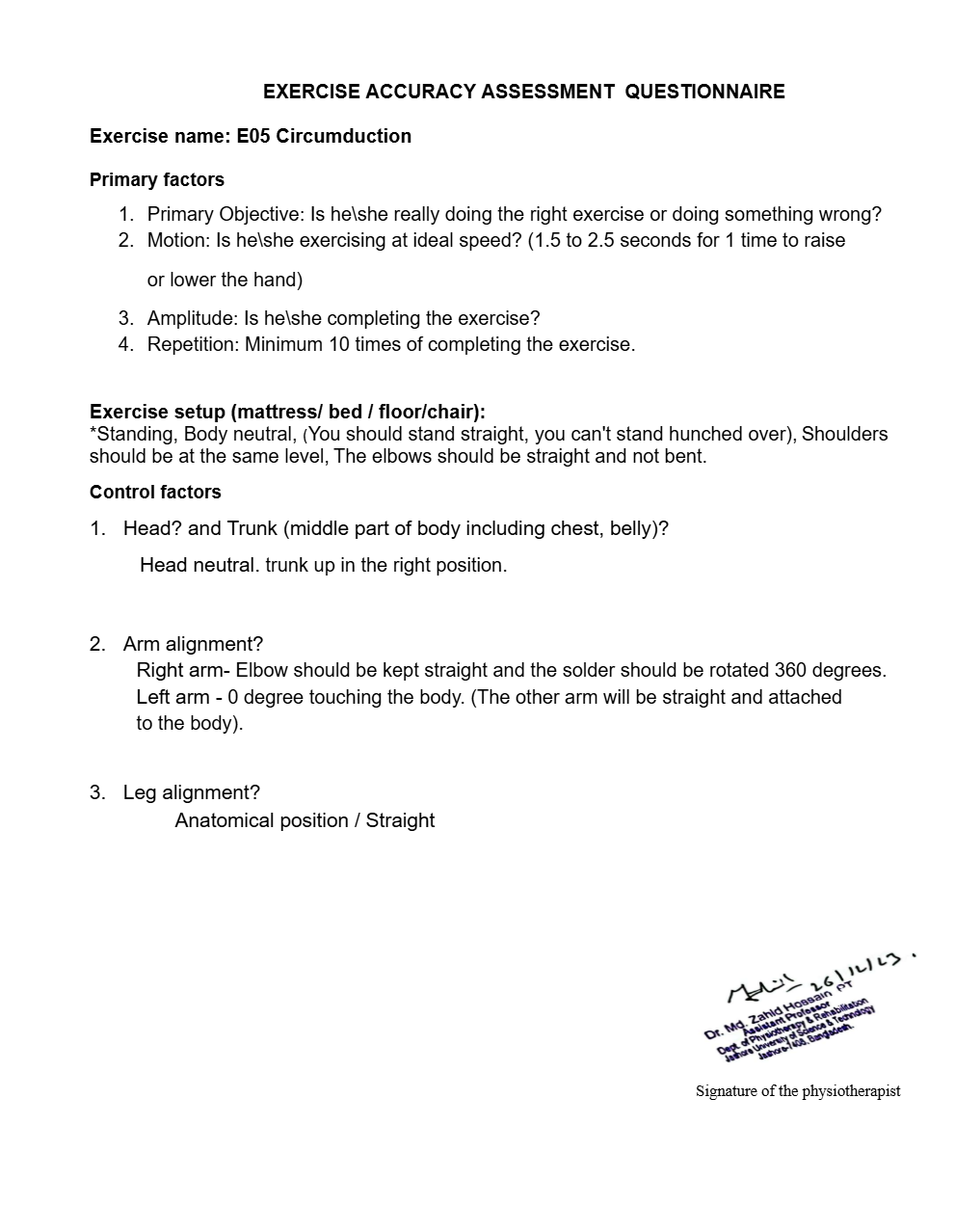 |
| Figure 12.e: Exercise Accuracy Assessment Questionnaire for Circumduction |
| 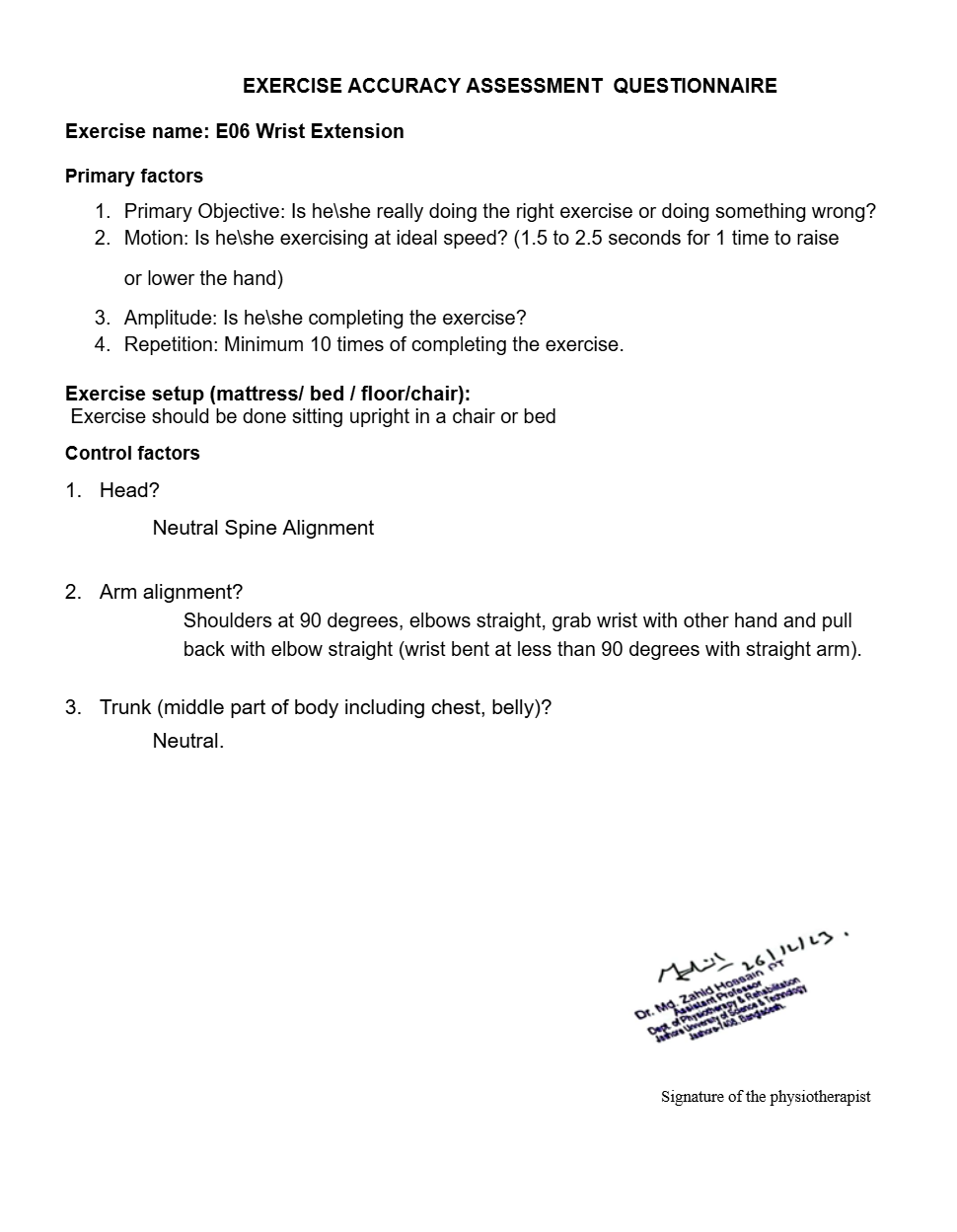 |
| Figure 12.f: Exercise Accuracy Assessment Questionnaire for Wrist Extension |
| 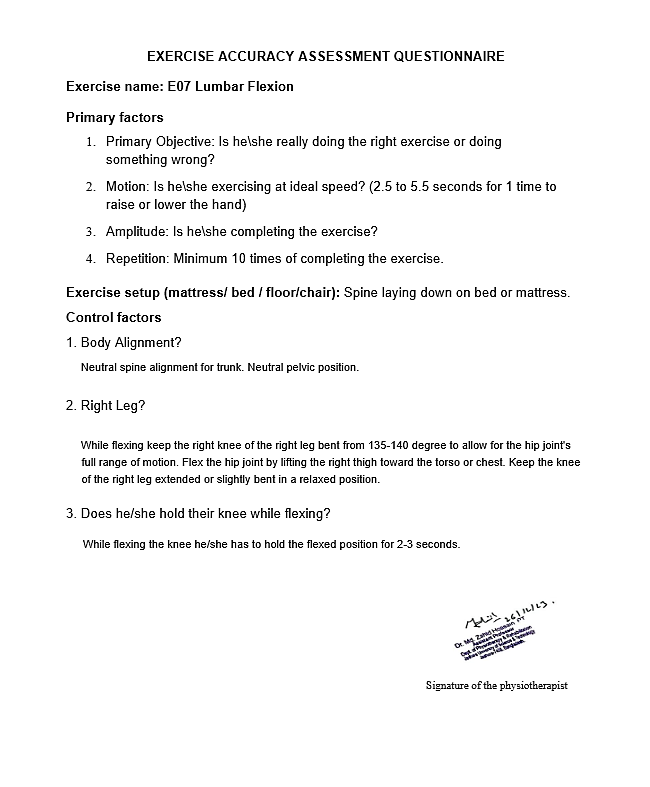 |
| Figure 12.g: Exercise Accuracy Assessment Questionnaire for A Lumbar Flexion |
| 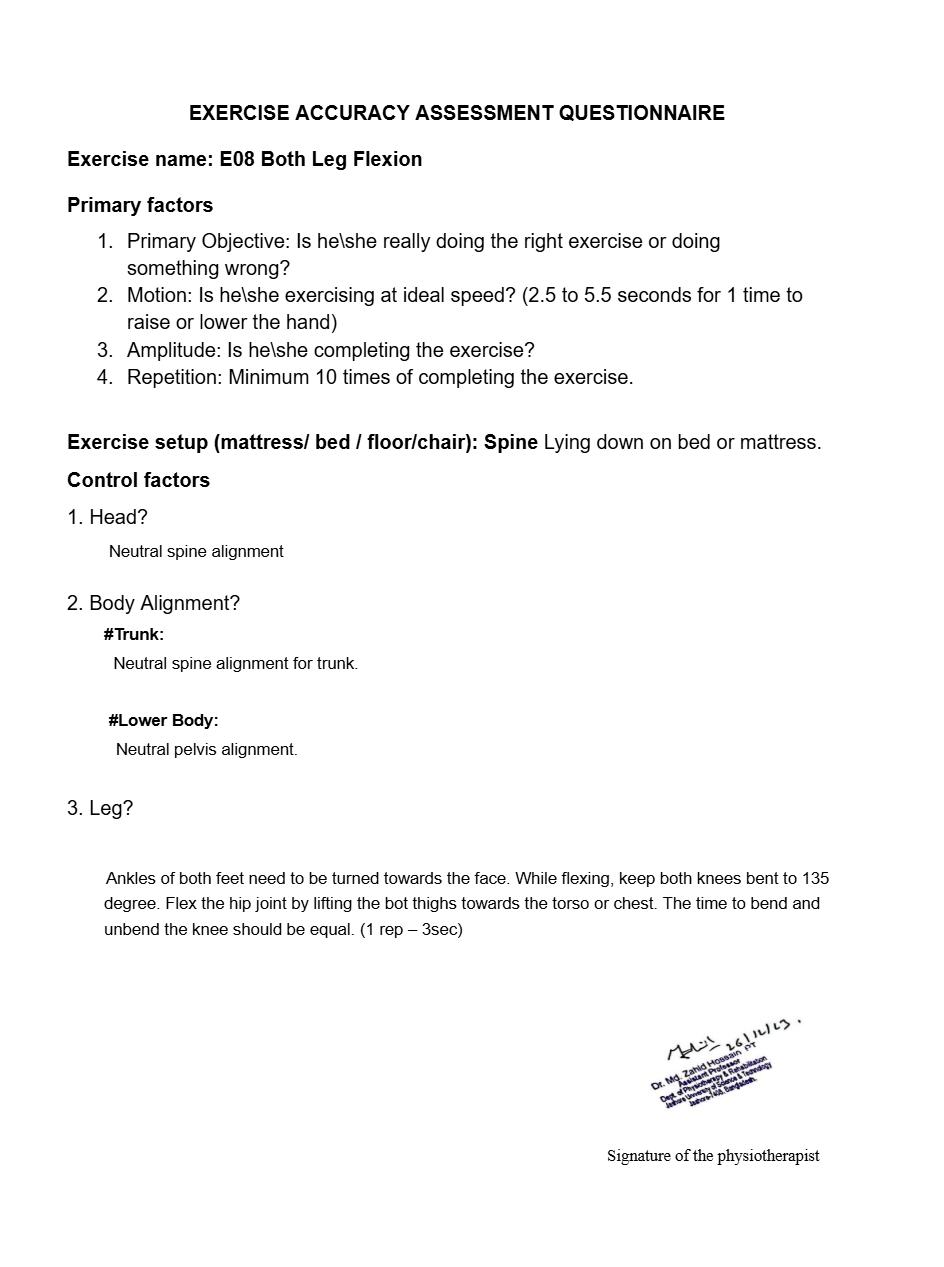 |
| Figure 12.h: Exercise Accuracy Assessment Questionnaire for Both Leg Flexion |
| 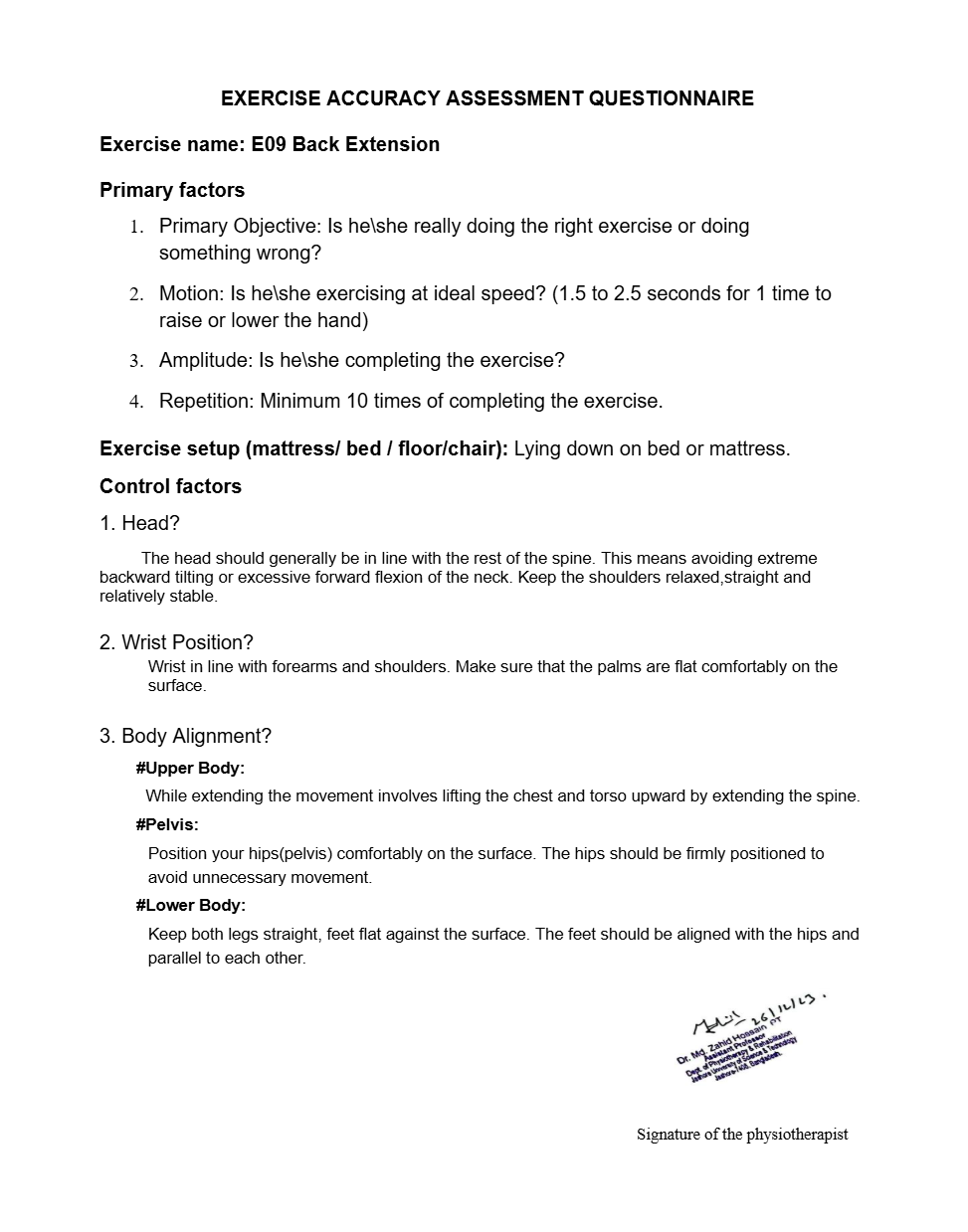 |
| Figure 12.i: Exercise Accuracy Assessment Questionnaire for Back Extension |
